# Supplementary material for: Antibody function predicts viral control in newborn monkeys immunised with an influenza virus HA stem nanoparticle
Source: Nat Commun. 2025 Apr 22;16:3785. doi: 10.1038/s41467-025-59149-8 (PMC12015251; doi:10.1038/s41467-025-59149-8)
Supplement: Supplementary file 1 — Supplementary Information [file 41467_2025_59149_MOESM1_ESM.pdf]

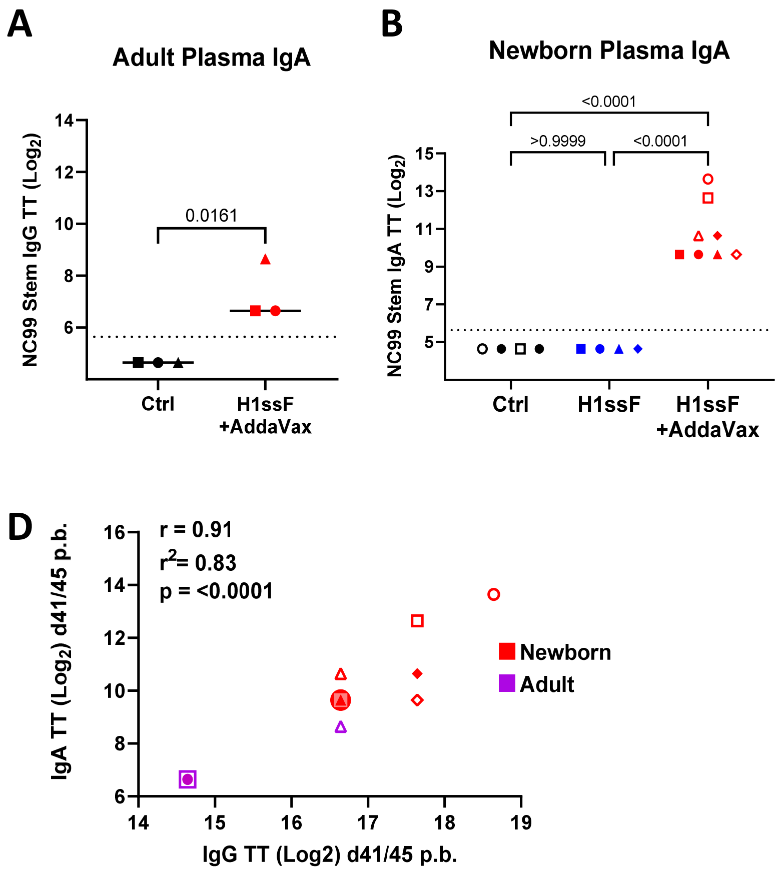

Supplementary Fig. 1. H1ssF+AddaVax vaccinated newborns have higher levels of circulating stem-specific IgA following prime/boost vaccination compared to vaccinated adults. Adult (A) and newborn (B) circulating levels of NC99 stem-specific IgA at d41/45 p.b. H1ssF+AddaVax newborn versus adult circulating stem-specific IgA TT (C). A Pearson's correlation was performed between stem-specific IgA TT (Log_2) and IgG TT (Log_2) at d41/45 p.b. in the H1ssF+AddaVax vaccinated infant (red symbols) and adult (pink symbols) AGMs (D). Ctrl adult (n=3), Ctrl newborn (n=5), H1ssF newborn (n=4), H1ssF+AddaVax adult (n=3), and H1ssF+AddaVax newborn (n=8). The dotted line represents the limit of detection (LOD) of the assay. Statistical significance was determined using a one-way ANOVA with Tukey's post hoc analysis (A) or a student's unpaired t test (B and C). Source data are provided as a Source Data file.

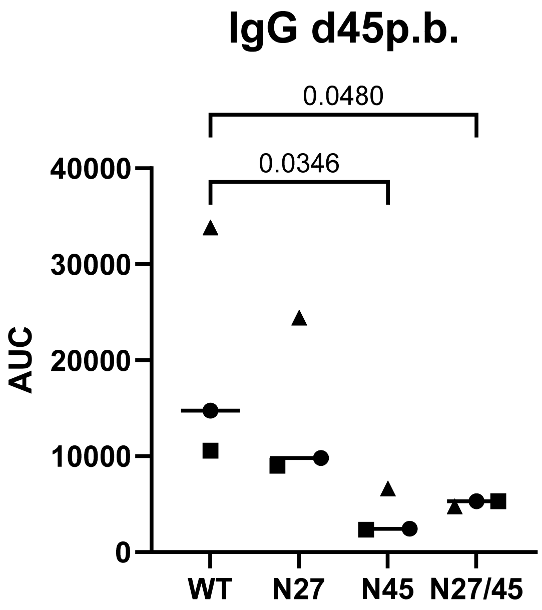

Supplementary Fig. 2. HA glycan mutant IgG levels in adult AGM. Circulating levels of IgG binding to full length NC99 HA WT, H1-N27, H1-N45, or H1-N27/45 were assessed by ELISA (AUC) at d45 p.b. in adult AGM. Ctrl (n=3), H1ssF+AddaVax (n=3). Animals are assigned a unique symbol that used throughout the study. Data represent the median. Statistical significance was determined using a one-way ANOVA with a Fisher's LSD post hoc analysis. Not significant p= >0.05 (not indicated on graph). Source data are provided as a Source Data file.

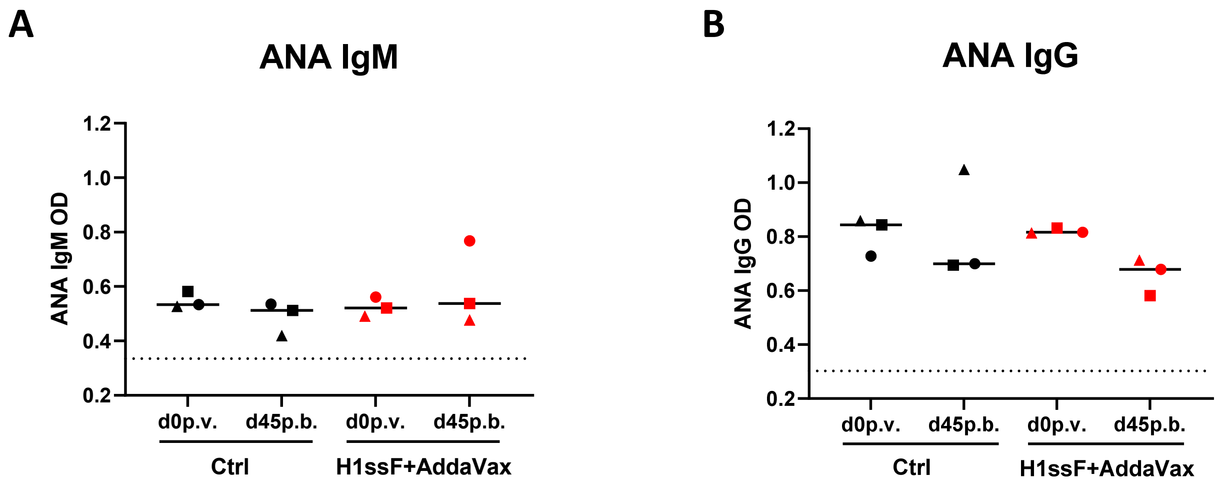

Supplementary Fig. 3. H1ssF+AddaVax does not promote increases in anti-nuclear Ab in adult AGM. Circulating levels of IgM (A) or IgG (B) binding to anti-nuclear Ab at d0 and d45 p.b. in adult AGM. Ctrl (n=3), H1ssF+AddaVax (n=3). Absorbance (OD) was measured at 450 nm. All plasma samples were diluted 1:50. The dotted line represents the limit of detection of the assay. Data represent the median. Statistical significance was determined using a one-way ANOVA with a Tukey's post hoc analysis. Not significant p= >0.05 (not indicated on graph). Source data are provided as a Source Data file.

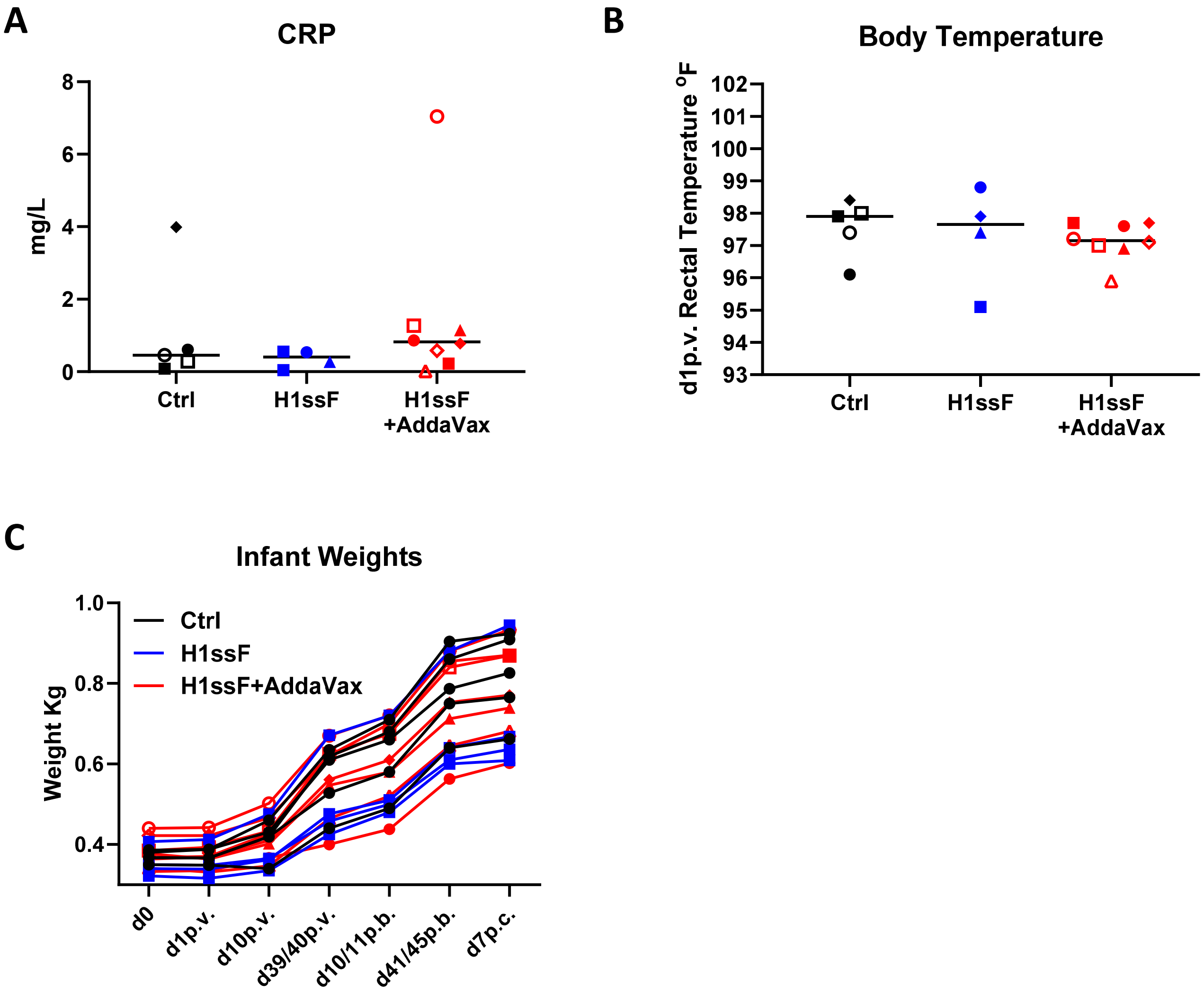

Supplementary Fig. 4. C-reactive protein levels, body temperatures, and weight gain were within normal ranges following H1ssF+AddaVax vaccination of newborn AGM. Circulating C-reactive protein levels (A) and rectal temperatures (B) were measured at d1p.v. in newborn AGMs to assess vaccine tolerability. Infant weights (kg) were measured over the course of the study (C). Ctrl (n=5), H1ssF (n=4), H1ssF+AddaVax (n=8). The line represents the median. Statistical significance was determined using a one-way ANOVA with a Fisher's LSD post hoc analysis. Not significant p= >0.05 (not indicated on graph). Source data are provided as a Source Data file.

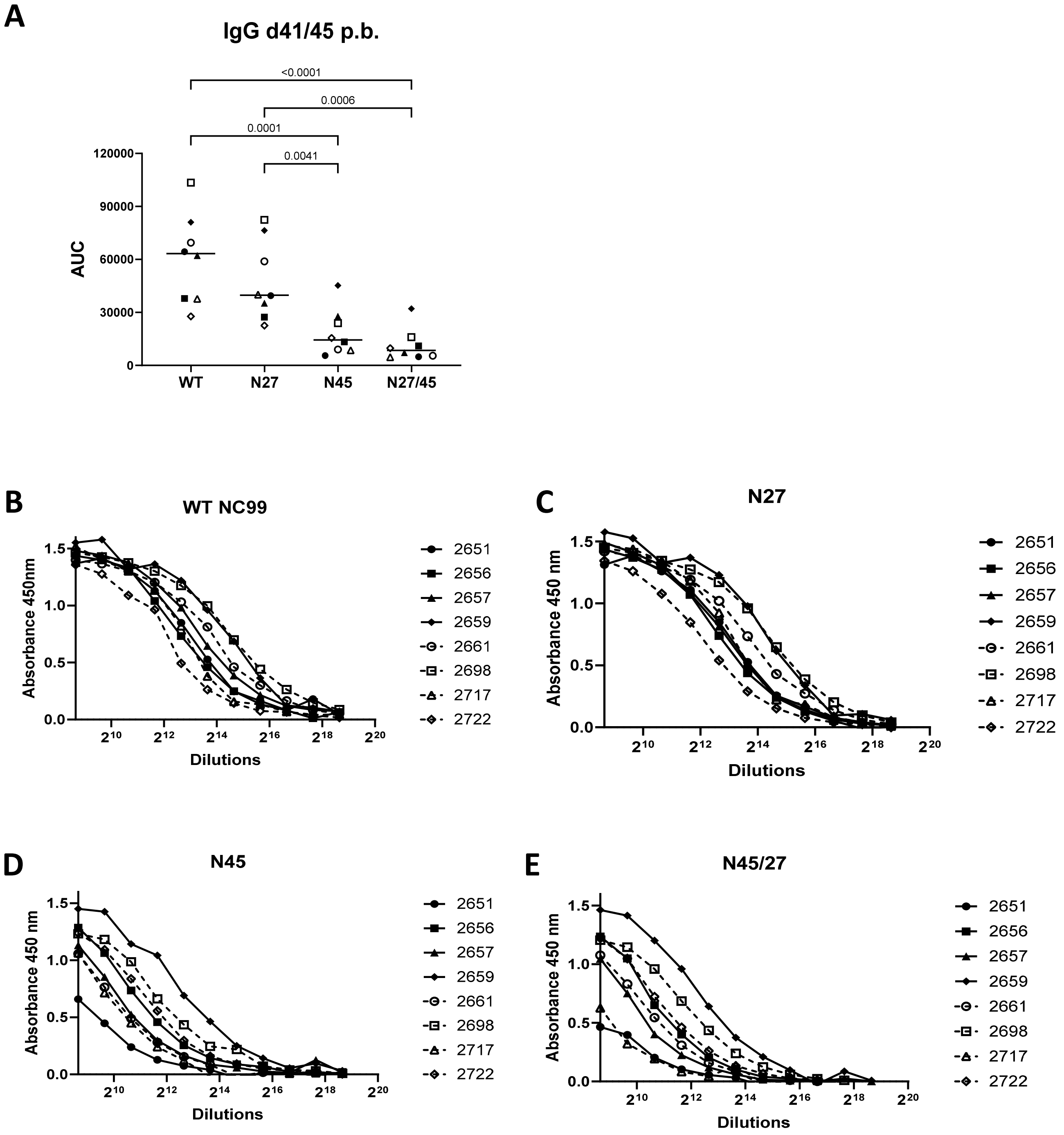

Supplementary Fig. 5. HA glycan mutant IgG levels in newborn AGM. Circulating levels of IgG binding to full length NC99 HA, H1-N27, H1-N45, or H1-N27/45 in newborn AGM vaccinated with H1ssF+AddaVax were measured by ELISA (AUC) at d41/45 p.b. (A). The OD_{450} at each dilution used to calculate the AUC for WT NC99 (B), N27 (C), N45 (D), and N45/27 (E). The line in A represents the median. Statistical significance was determined using a one-way ANOVA with a Fisher's LSD post hoc analysis. Not significant p= >0.05 (not indicated on graph). Source data are provided as a Source Data file.

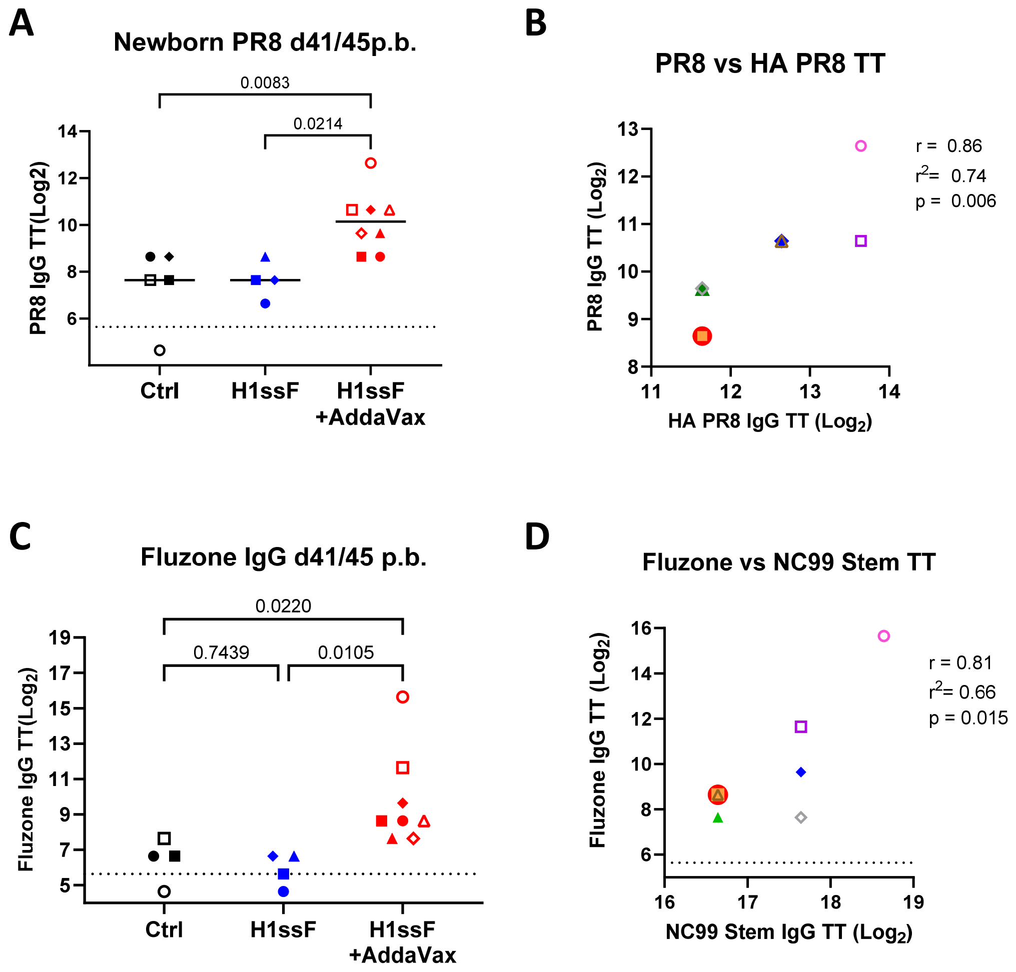

Supplementary Fig. 6. Antibodies generated by newborn vaccination with H1ssF+AddaVax recognize stem presented in the context of an influenza virion and 2023-2024 Fluzone. The ability of Ab elicited by H1ssF+AddaVax vaccination to bind PR8 virus was determined by ELISA at d41/45 p.b (A). A Pearson correlation analysis was performed on the IgG TT (Log2) measured for PR8 virus and PR8 HA protein (B). IgG specific for the Fluzone vaccine from 2023-2024 season was measured in plasma at d41/45 p.b. (C). A Pearson correlation analysis was performed using the IgG TT (Log2) measured in H1ssF+AddaVax vaccinated newborns for Fluzone versus NC99 stem (D). The line in A represents the median. Statistical significance was determined using a one-way ANOVA with a Tukey's post hoc analysis. Not significant p= >0.05 (not indicated on graph). Source data are provided as a Source Data file.

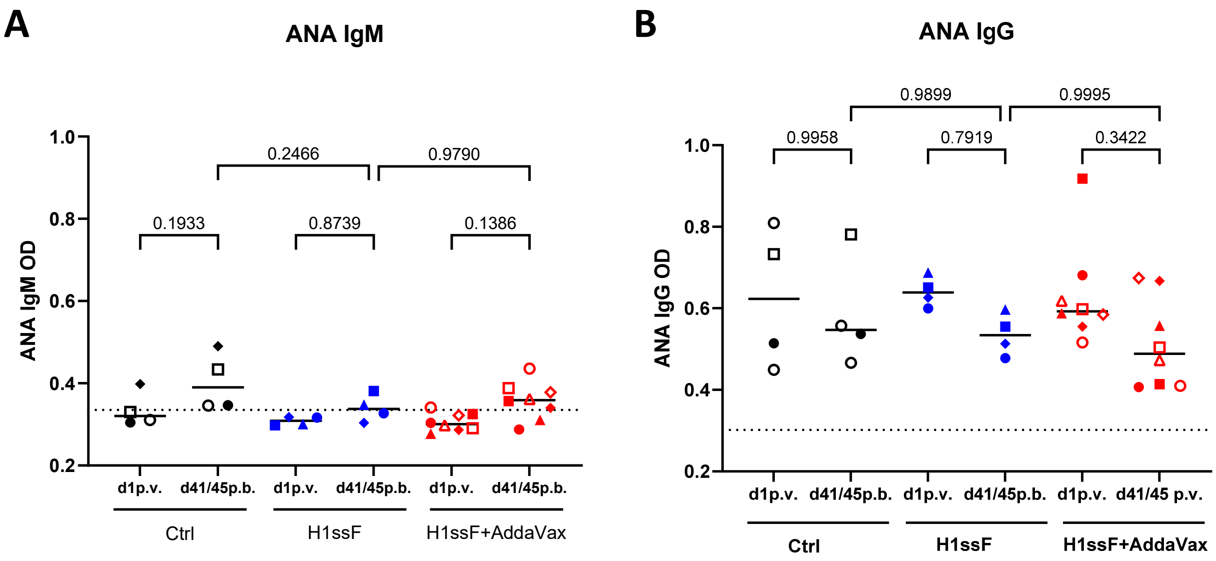

Supplementary Fig. 7. H1ssF+AddaVax does not promote increases in anti-nuclear Ab in newborn AGM. Circulating levels of IgM (A) or IgG (B) binding to anti-nuclear Ab at d1 p.v. and d41/45 p.b. in newborn AGM. All plasma samples were diluted 1:50. The dotted line represents the limit of detection of the assay. The line represents the median. Statistical significance was determined using a one-way ANOVA with a Tukey's post hoc analysis. p= >0.05 considered not significant. Source data are provided as a Source Data file.

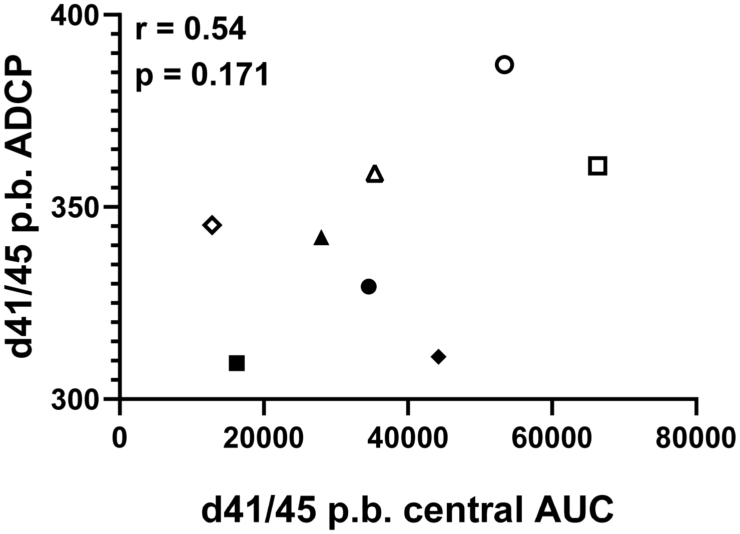

Supplementary Fig. 8. No significant correlation is observed between ADCP activity and IgG Abs specific for the central stem epitope at d41/45 p.b. A Spearman's correlation analysis was performed using ADCP activity and central stem epitope IgG AUC at d41/45 p.b. in H1ssF+AddaVax vaccinated infant AGMs. Source data are provided as a Source Data file.

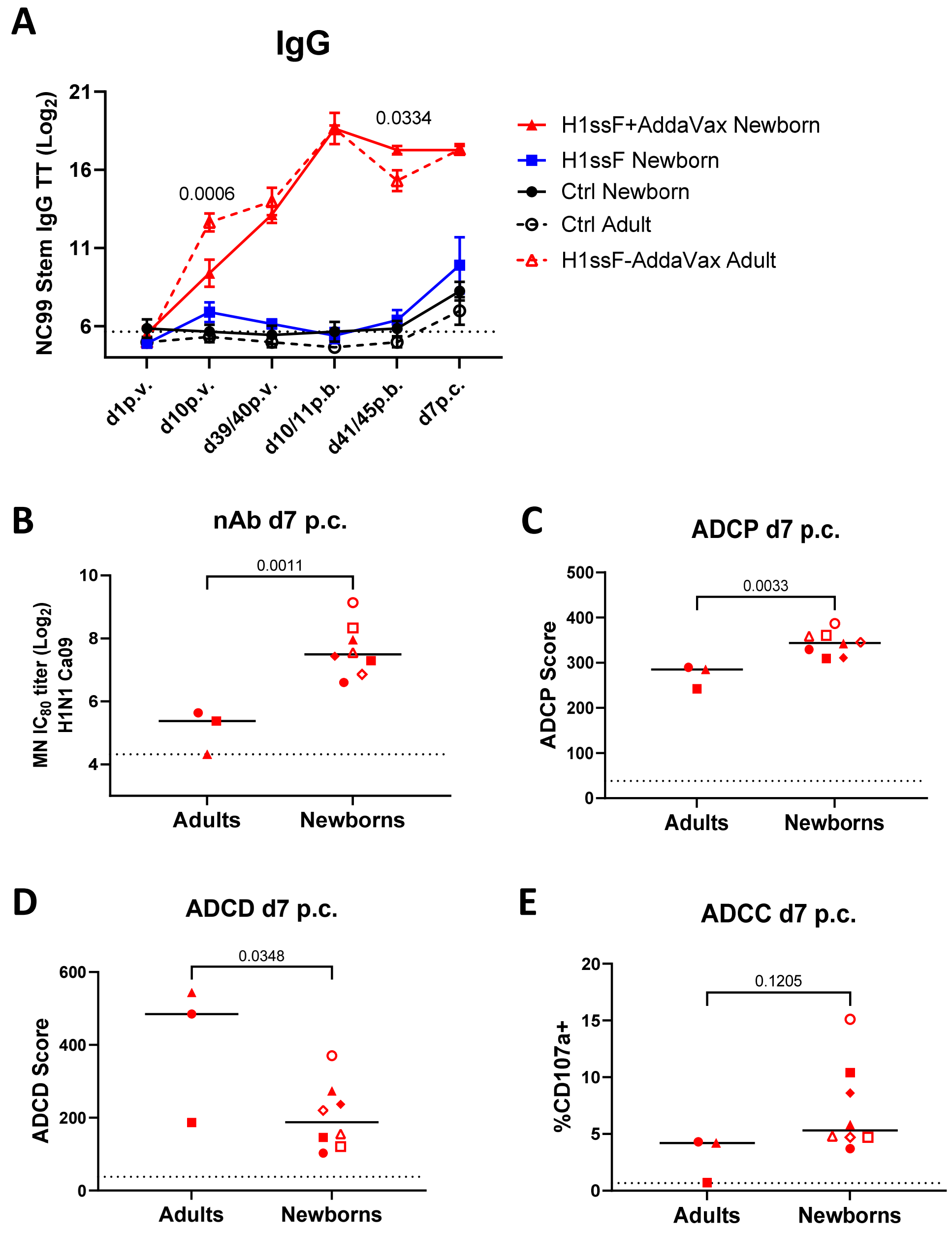

Supplementary Fig. 9. Newborn and adult animals administered H1ssF+AddaVax differ in the quality of the Ab response present following Ca09 challenge. NC99 stem-specific IgG in adult and newborn animals (A). Neutralizing Ab IC_{80} titers against H1N1 A/New Caledonia/20/1999 (B), ADCP (C), ADCD (D), and ADCC activity (E) were measured at d7 p.c. in Ctrl adult (n=3), Ctrl newborn (n=5), H1ssF newborn (n=4), H1ssF+AddaVax adult (n=3), and H1ssF+AddaVax newborn (n=8). The dotted line represents the limit of detection (LOD) of the assay. The line represents the mean ±SEM (A) or median (B, C, D, and E). Statistical significance was determined using a two-way ANOVA with a Tukey's post hoc analysis (A), or a student's unpaired t test (B, C, D, and E). Not significant p = >0.05. Source data are provided as a Source Data file.

Supplementary Table 1. Adult AGM enrollment information and identifiers

Animal I.D	Vaccine Received	Sex	Age 1st dose (Years)	Symbol
2379	PBS	M	5.76	●
2405	PBS	M	5.49	■
2416	PBS	M	5.38	◆
2368	H1ssF+AddaVax	M	6.12	●
2402	H1ssF+AddaVax	M	5.52	■
2412	H1ssF+AddaVax	M	5.42	▲

Supplementary Table 2. Newborn AGM enrollment data and identifiers

Animal I.D	Vaccine Received	Sex	Age 1st dose (Days)	Symbol
2644	PBS	M	3	● (black)
2664	PBS	F	3	■ (black)
2808	PBS	M	4	◆ (black)
2737	mRNA Luciferase-LNP	M	5	○ (black)
2785	mRNA Luciferase-LNP	F	4	□ (black)
2720	H1ssF	M	3	● (blue)
2723	H1ssF	M	4	■ (blue)
2729	H1ssF	F	5	▲ (blue)
2741	H1ssF	F	3	◆ (blue)
2651	H1ssF+AddaVax	F	5	● (red)
2656	H1ssF+AddaVax	M	3	■ (red)
2657	H1ssF+AddaVax	F	3	▲ (red)
2659	H1ssF+AddaVax	M	4	◆ (red)
2661	H1ssF+AddaVax	F	5	○ (red)
2698	H1ssF+AddaVax	M	5	□ (red)
2717	H1ssF+AddaVax	M	5	△ (red)
2722	H1ssF+AddaVax	F	4	◇ (red)

Supplementary Table 3. Antibodies used in study

Antibody	Clone	Dilution
Anti-monkey IgM-HRP	Fitzgerald #43R-IG074hrp	1:10,000
Anti-monkey IgG-HRP	Fitzgerald #43C-CB1603	1:5,000
Anti-monkey IgA-Biotin	Fitzgerald 43R-IG002bt	1:5,000
Anti-CD107a-PE	Biolegend, #328608	1:50
Anti-guinea pig C3-FITC	MP Biomedicals, # 855385	1:100

Supplementary Table 4. PCR primers and target sequence

CDC designed primer sequences used for RT-qPCR

InfA For1 5′-CAAGACCAATCYTGTCACCTCTGAC-3′
InfA For2 5′-CAAGACCAATYCTGTCACCTYTGAC-3′
InfA Rev1 5′-GCATTYTGGACAAAVCGTCTACG-3′
InfA Rev2 5′-GCATTTTGGATAAAGCGTCTACG-3′
InfA Probe 5′-/5FAM/ TGCAGTCCT/ZEN/CGCTCACTGGGCACG/3IABkFQ/-3′

Synthetic DNA target sequence:

cgactaatacgactcactatagggagaCAAGACCAATCTTGTCACCTCTGACTAAGGGAATTTTAGGATTTGT
GTTCACGCTCACCGTGCCCAGTGAGCGAGGACTGCAGCGTAGACGCTTTATCCAAAATGCaac
atttacagcTCCTCAACTCACTCTTCGAGCGTCTCAATGAAGGACATTCAAAGCCAATTCGAGCAG
CTGAAACTGCGGTGGGAGTCTTATCCCAATTTGGTCAAGAGCACCGCttctatagtgtcacctaaatggatct
