# Supplementary material for: Antibody function predicts viral control in newborn monkeys immunised with an influenza virus HA stem nanoparticle
Source: Nat Commun. 2025 Apr 22;16:3785. doi: 10.1038/s41467-025-59149-8 (PMC12015251; doi:10.1038/s41467-025-59149-8)
Supplement: Supplementary file 2 — Reporting Summary [file 41467_2025_59149_MOESM2_ESM.pdf]

nature portfolio

Corresponding author(s): Martha Alexander-Miller

Last updated by author(s): Apr 1, 2025

Reporting Summary

Nature Portfolio wishes to improve the reproducibility of the work that we publish. This form provides structure for consistency and transparency in reporting. For further information on Nature Portfolio policies, see our Editorial Policies and the Editorial Policy Checklist.

Statistics

For all statistical analyses, confirm that the following items are present in the figure legend, table legend, main text, or Methods section.

n/a	Confirmed	
☐	☒	The exact sample size (*n*) for each experimental group/condition, given as a discrete number and unit of measurement
☐	☒	A statement on whether measurements were taken from distinct samples or whether the same sample was measured repeatedly
☐	☒	The statistical test(s) used AND whether they are one- or two-sided<br>*Only common tests should be described solely by name; describe more complex techniques in the Methods section.*
☒	☐	A description of all covariates tested
☐	☒	A description of any assumptions or corrections, such as tests of normality and adjustment for multiple comparisons
☐	☒	A full description of the statistical parameters including central tendency (e.g. means) or other basic estimates (e.g. regression coefficient) AND variation (e.g. standard deviation) or associated estimates of uncertainty (e.g. confidence intervals)
☐	☒	For null hypothesis testing, the test statistic (e.g. *F*, *t*, *r*) with confidence intervals, effect sizes, degrees of freedom and *P* value noted<br>*Give P values as exact values whenever suitable.*
☒	☐	For Bayesian analysis, information on the choice of priors and Markov chain Monte Carlo settings
☒	☐	For hierarchical and complex designs, identification of the appropriate level for tests and full reporting of outcomes
☐	☒	Estimates of effect sizes (e.g. Cohen's *d*, Pearson's *r*), indicating how they were calculated

Our web collection on statistics for biologists contains articles on many of the points above.

Software and code

Policy information about availability of computer code

Data collection	BD FACS Diva V9, BioTek Gen5
Data analysis	BD FACS Diva V9, GraphPad Prism 10.4, SAS V9.4

For manuscripts utilizing custom algorithms or software that are central to the research but not yet described in published literature, software must be made available to editors and reviewers. We strongly encourage code deposition in a community repository (e.g. GitHub). See the Nature Portfolio guidelines for submitting code & software for further information.

Data

Policy information about availability of data

All manuscripts must include a data availability statement. This statement should provide the following information, where applicable:

- Accession codes, unique identifiers, or web links for publicly available datasets
- A description of any restrictions on data availability
- For clinical datasets or third party data, please ensure that the statement adheres to our policy

Primary data are included in the source data file submitted with the manuscript.

Human research participants

Policy information about studies involving human research participants and Sex and Gender in Research.

Reporting on sex and gender	NA
Population characteristics	NA
Recruitment	NA
Ethics oversight	NA

Note that full information on the approval of the study protocol must also be provided in the manuscript.

Field-specific reporting

Please select the one below that is the best fit for your research. If you are not sure, read the appropriate sections before making your selection.

- [x] Life sciences
- [ ] Behavioural & social sciences
- [ ] Ecological, evolutionary & environmental sciences

For a reference copy of the document with all sections, see nature.com/documents/nr-reporting-summary-flat.pdf

Life sciences study design

All studies must disclose on these points even when the disclosure is negative.

Sample size	Each group contained 8 animals (4 male and 4 female). Using PASS 13 software we determined that in a repeated measures design with 8 animals measured at 4 post-treatment time points there is 80% power to detect a difference of 1.16 standard deviations (SD) between groups for an outcome of interest using a compound symmetry covariance structure and a correlation between repeated measures of 0.58 (based on our previous work) with alpha=0.05 (2-sided). Based on our previous data, the estimated SD for IgG was at most 1.5 and the correlation between repeated observations was 0.58. Using these inputs we can detect a difference in IgG value of 1.74 titer units with 80% power and alpha=0.05 (2-sided test). For analyses that may focus on one timepoint, we would have 80% power to detect a difference between groups of 2.26 titer units based on our estimate of the variability in IgG measures.
Data exclusions	Data are not excluded.
Replication	Findings are replicated with individuals animals.
Randomization	Newborns were randomized to the experimental groups.
Blinding	Blinding was not possible as the animals were numbered by sample and analyzed by researchers involved in the study.

Reporting for specific materials, systems and methods

We require information from authors about some types of materials, experimental systems and methods used in many studies. Here, indicate whether each material, system or method listed is relevant to your study. If you are not sure if a list item applies to your research, read the appropriate section before selecting a response.

Materials & experimental systems

n/a	Involved in the study	
☐	☒	Antibodies
☐	☒	Eukaryotic cell lines
☒	☐	Palaeontology and archaeology
☐	☒	Animals and other organisms
☒	☐	Clinical data
☒	☐	Dual use research of concern

Methods

n/a	Involved in the study	
☒	☐	ChIP-seq
☒	☐	Flow cytometry
☒	☐	MRI-based neuroimaging

Antibodies

Antibodies used	Anti-monkey IgM-HRP Fitzgerald #43R-IG074hrp 1:10,000<br>Anti-monkey IgG-HRP Fitzgerald #43C-CB1603 1:5,000<br>Anti-monkey IgA-Biotin Fitzgerald 43R-IG002bt 1:5,000

	Anti-CD107a-PE Biolegend, #328608 1:50<br>Anti-guinea pig C3-FITC MP Biomedicals, # 855385 1:100
Validation	Antibodies were validated by the vendor.

Eukaryotic cell lines

Policy information about cell lines and Sex and Gender in Research

Cell line source(s)	THP-1 cells were obtained from the WFUSM CCC Core via ATCC (lot number #70035863), KHYG-1 cells transduced with rhesus macaque CD16 (kind gift of Dr. David Evans)
Authentication	THP-1 cells were authenticated by the vendor. CD16 expression of KHYG-1 ells was tested for flow cytometry.
Mycoplasma contamination	The cell lines were not tested for mycoplasma just prior to the analysis.
Commonly misidentified lines<br>(See ICLAC register)	NA

Animals and other research organisms

Policy information about studies involving animals; ARRIVE guidelines recommended for reporting animal research, and Sex and Gender in Research

Laboratory animals	African green monkeys were from the breeding colony at Wake Forest University School of Medicine.
Wild animals	NA
Reporting on sex	Equal numbers of male and female animals were used. Sex was considered in the results. No sex dependent differences were observed.
Field-collected samples	NA
Ethics oversight	The animal care and use protocol was adherent to the US Animal Welfare Act and Regulations and approved by the Wake Forest University Institutional Animal Care and Use Committee. AGM were housed and cared for in accordance with state, federal, and institute policies in facilities accredited by the American Association for Accreditation of Laboratory Animal Care (AAALAC) under standards established in the Animal Welfare Act and the Guide for the Care and Use of Laboratory Animals.

Note that full information on the approval of the study protocol must also be provided in the manuscript.
